# The Treatment of Abortion and Some of the Complications Incident Thereto

**Published:** 1892-07

**Authors:** Walter A. Crow

**Affiliations:** Atlanta, Ga.


### THE TREATMENT OF ABORTION AND SOME OF THE COMPLICATIONS INCIDENT THERETO.*

BY

WALTER A. CROW, M. D.,

ATLANTA, GA.

Dr. Crow said that this was a condition, the results of which give rise to the necessity of special treatment in the hands of the gynecologist and abdominal surgeon more than any other single cause in the diseases of the female human subject. From the physiological changes of thickening and hypertrophy of the muscularæ, also the changes of the endometrium, as claimed by Johnstone and others as to it being classified as an adenoid tissue; and during gestation that this uterine lymphatic system undergoes hypertrophic and hyperplastic changes in all form elements, analogous in kind and degree to those of the blood vessels; that it thereby favored the tendency to septic infection in all cases of abortion. The mildest type, but the most common form, was the arrest of the retrograde involution of the uterus, a condition known as subinvolution, and from this other and more pronounced types

*An abstract of a paper read at the Georgia Medical Association, April, 1892.

were met with, such as pyosalpinx, abscess of the ovaries, pelvic or general peritonitis; also the marked tendency to recurrent attacks of an inflammatory character, either acute or sub-acute. Under this he mentions metritis, chronic endometritis, or an extension of the trouble so as to involve the appendages, also the adjacent tissue and vicera. Malpositions, especially the backward displacement, was a most common occurrence.

The plan of treatment is as near aseptic as the surrounding circumstances will admit of.

He first has the patient take a sitz bath with the liberal use of soap; or if this cannot be done, then bathe the parts with a cloth or sponge. This is then followed with a one to 2,000 or 5,000 bichloride solution in vagina. He favors plugging the cervix to rapid dilatation unless there are special indications for immediate delivery. After the fœtal contents have been removed, he insists in all cases of thoroughly satisfying one's self as to the exact condition of the interior of the womb, thereby avoiding the possible risk of a retained piece of placenta or strip of membrane. To do this he suggests the use of a small wire curette, which, with a little practice and observation, will enable any one to form a very accurate idea of the endometrium. After the uterus has been cleared, he again uses the same strength of the bichloride solution, and follows the routine treatment that is common in full term delivery.

In the treatment of the after troubles he relates three cases illustrative:

CASE I was one in which a portion of the placenta was retained, and followed some four months afterwards with a profuse hemorrhage, also a condition of subinvolution with some tubal trouble. This was treated with curettement and packing the uterus with iodoform gauze, opening the bowelswell with salines so as to relieve a tendency to pelvic engorgement, also a tonic iron mixture.

CASE II was one of subinvolution retroversion, also a pyosalpinx on left side. A laparotomy was advised in this case, but refused. The next best thing was to dilate and favor drainage. This patient showed a marked improvement but was not cured. She is again pregnant.

CASE III.—Case of abortion six years ago; has suffered more or less from uterine troubles since. Condition when called to see her eighteen months ago was chronic endometritis, retroflexion with a profuse menorrhagia. This condition, complicated with la grippe, rendered her condition a serious one. But after a thorough curetting and prolonged after treatment of local applications, also the use of oxygen gas and the most favorable surroundings, she was improved very much, but still has a misplaced uterus slightly enlarged.

The summary of the paper is as follows:

1. The marked tendency to infection in all cases of abortion.

2. The urgent necessity of removing as far as possible all sources of infection. This includes clean hands, clean instruments, cleanliness of person and bedding.

3. The importance of thoroughly satisfying one's self in all cases of the complete removal of all the fœtal membrane and placental tissue.

4. Keep your patient under observation for at least two months, with the most favorable surroundings for complete and perfect involution of the enlarged uterus, building up the general health and keeping the bowels open, if necessary, with salines.

5. Be urgent in your advice in avoiding the possibility of becoming pregnant for at least six months, as sexual connection produces more or less irritation and congestion of the parts, and thereby retards, if not in a measure stops, the condition we most desire in these cases—a normal restoration of the parts.

